# Maintenance and inspection as risk factors in helicopter accidents: Analysis and recommendations

**DOI:** 10.1371/journal.pone.0211424

**Published:** 2019-02-01

**Authors:** Joseph Homer Saleh, Archana Tikayat Ray, Katherine S. Zhang, Jared S. Churchwell

**Affiliations:** School of Aerospace Engineering, Georgia Institute of Technology, Atlanta, GA, United States of America; University of Louisville, UNITED STATES

## Abstract

In this work, we establish that maintenance and inspection are a risk factor in helicopter accidents. Between 2005 and 2015, flawed maintenance and inspection were causal factors in 14% to 21% of helicopter accidents in the U.S. civil fleet. For these maintenance-related accidents, we examined the incubation time from when the maintenance error was committed to the time when it resulted in an accident. We found a significant clustering of maintenance accidents within a short number of flight-hours after maintenance was performed. Of these accidents, 31% of these accidents occurred within the first 10 flight-hours. This is reminiscent of infant mortality in reliability engineering, and we characterized it as *maintenance error infant mortality*. The last quartile of maintenance-related accidents occurred after 60 flight-hours following maintenance and inspection. We then examined the “physics of failures” underlying maintenance-related accidents and analyzed the prevalence of different types of maintenance errors in helicopter accidents. We found, for instance, that the *improper or incomplete (re)assembly or installation of a part* category accounted for the majority of maintenance errors with 57% of such cases, and within this category, the incorrect torquing of the B-nut and incomplete assembly of critical linkages were the most prevalent maintenance errors. We also found that within the *failure to perform a required preventive maintenance and inspection task* category, the majority of the maintenance programs were not executed in compliance with federal regulations, nor with the manufacturer maintenance plan. Maintenance-related accidents are particularly hurtful for the rotorcraft community, and they can be eliminated. This is a reachable objective when technical competence meets organizational proficiency and the collective will of all the stakeholders in this community. We conclude with a set of recommendations based on our findings, which borrow from the ideas underlying the defense-in-depth safety principle to address this disquieting problem.

## Introduction

The fundamental feature of a helicopter’s design, a *rotating wing*, decouples the vehicle airspeed from that experienced by the airfoil or blade element on its main rotor. As a result, unlike the situation with *fixed-wing* aircraft, lift generation is not contingent on the forward motion of the vehicle. The consequence of this is that several new flight regimes are enabled by this particular design choice, including hovering, vertical flight, and translational flight in directions (e.g., backward, and sideways) that are beyond the flight envelope of *fixed wings*. These flight regimes have made the helicopter indispensable for many civilian and military applications, from medical evacuation to law enforcement and close air support to mention a few.

By the same token however, *rotating the wing* raises a host of design and operational challenges and complexities, including issues related to vibrations and wear-out of a variety of components on the helicopter. The consequence of this is that maintenance and inspection are particularly crucial for helicopters for their continued airworthiness. In this work, we examine helicopter maintenance and inspection and their association with helicopter accidents. To place this work in proper context, some background is warranted.

Helicopters have a poor safety track record and little progress has been recorded in the last decade, at least in their fatal accident rates. The present work is part of a larger effort that focuses on the epidemiology of helicopter accidents. Its aim is to provide a better understanding of helicopter accidents and to identify possible blind spots and important areas for different stakeholders to focus their attention and resources for accident prevention. The end-objective is to contribute toward improving the safety track record of helicopters, and ultimately to reduce the burden of injuries, fatalities, and financial losses due to helicopter accidents. In a previous work, we examined trends, rates, and factors associated with helicopters accidents [1]. We controlled for number of main rotor blades, engine type (turboshaft versus reciprocating), and number of engines (single versus twin). The key findings of that study are briefly summarized below:

1. At an aggregate level, we found that the fatal accident rate of U.S. civil helicopters has averaged 0.7 fatal accidents per 100,000 flight-hours since 2005, and no statistically significant improvement has occurred since then. Similarly, we found that the total accident rate has averaged about 4.8 accidents per 100,000 flight-hours;
2. Helicopters have a 17.3 times or 1,730% the risk of fatal accidents of passenger cars in the U.S. (based on the size of the respective fleets). This is a staggering result and it provides a benchmark for appreciating the safety record of helicopters;
3. Helicopter accident rates vary by number of main rotor blades, by engine type, and by number of engines (when controlling for each of the other factors). For instance, the 2-bladed (2B) reciprocating helicopters have an accident rate 1.31 times that of the 2B turboshaft. This risk ratio increases to 1.56 when comparing the 3B reciprocating with the 3B turboshaft helicopters;

We noted that these findings deserve careful attention and can help operators and regulators better target their prevention efforts for more effective safety interventions. Additional details can be found in [1].

In order to improve the safety record of helicopters, it is important to understand the various causes and failure mechanisms leading to their accidents. Different studies have consistently ascribed 60% to 70% of all helicopter accidents to *pilot errors*, and most of the rest—*unknown* causes excluded—to *airworthiness* causes, which include failure through fatigue, corrosion, or wear of components such as the power plant, rotor, or transmission [2].

What has not been investigated are the contributions of maintenance and inspection to helicopter accidents, if any. This is the focus of the present work. The topic may sound counter-intuitive, and we began our investigation asking a more benign question: we wanted to examine and characterize the *time to accident* of helicopters after maintenance and inspection. Our preliminary results led us on a different path and raised a number of others questions, the answers to which identified maintenance and inspection as risk factors in helicopter accidents. *Risk factor* is a term epidemiologists typically use instead of *cause*, e.g., asbestos as a risk factor in cancer development. This reflects in part their cautiousness about making causal inferences, especially when working with observational studies, not experimental studies or randomized control trials [3].

What began as a simple statistical exercise led us to a sobering find, that maintenance and inspections are associated with, and in some cases direct causes of helicopter accidents. This result points to an important blind spot for the rotorcraft community to direct their attention to in order to improve helicopter safety, even if by a small increment. Maintenance and inspection related accidents are particularly hurtful for the community, and they cannot be addressed if not acknowledged and discussed. The present work identifies and provides a careful examination of this issue, and we hope it will encourage other authors and organizations to investigate this problem further with the end-objective of eliminating it.

The remainder of this work is organized as follows. In section 2, we discuss the data sources and methods used in this work, as well as some the regulations that cover rotorcraft maintenance and inspection. In section 3, the core of this work, we examine different aspects of the association between maintenance and inspection and helicopter accidents. In section 4, we propose a set of recommendations for addressing maintenance errors based on our previous findings, which borrow from the ideas underlying the defense-in-depth safety principle. Finally, we conclude this work in Section 5.

## Brief literature review

This work is at the intersection of two broad themes: maintenance errors on the one hand and helicopter accidents on the other. In this section, we briefly review both themes to provide the background and context for our work.

Human factors in accidents have traditionally focused on operator or pilot errors, and there is an extensive literature on this subject. This body of work is not within the scope of the present work. A smaller but more recent concern has shifted the focus from operators and pilots, and instead examined human factors in maintenance, maintenance errors, and their role in industrial accidents. The two ways of looking at the subject have been: (1) the industrial process and the maintenance operation as a hazard to the maintainers (injuries and death of the individuals performing maintenance); (2) the maintainers performing inadequate work, and as as a result causing or contributing to future equipment failures or industrial accidents. Lind [4], for example, examined causes and types of accidents to individuals while they were performing industrial maintenance operations. She identified the most common types of accidents to maintainers and the underlying reasons for these occurrences, from unsafe acts and dangerous working conditions to defective machinery and insufficient experience while performing said maintenance. A broad review of human error in maintenance across different industries is provided in Dhillon and Liu [5]. Their main finding is that while maintenance plays an important role in equipment reliability, human error in maintenance is an under-recognized but pressing safety concern. Several works have narrowed this focus to the airline industry and examined human factors in aircraft maintenance. For example, Gramopadhye and Drury [6] provided a broad theoretical framework for human factors in aviation maintenance. They made the case for the need of such focus and how to address it. Krulak [7] examined the association between maintenance errors and aircraft mishap frequency and severity. The author lamented the “dearth of information relating human-factors in maintenance related accidents” He found, for example, that the most common factors involved in maintenance-related mishaps were inadequate supervision, decision errors, attention errors, and inadequate maintenance processes.

Helicopter accidents have generally not received the same level of attention from the media or in the safety literature, nor have they been examined with the same level of thoroughness as accidents in the chemical or airline industries, as we have discussed in the companion article [1]. The studies have generally focused on helicopter accidents by mission type, e.g., helicopter emergency medical services or HEMS [8, 9], or accidents in a particular area, for example in the U.K. [10] or in Hawaii [11]. Other studies have examined safety of helicopter operations associated with offshore Oil and Gas operations in the North Sea for example [12, 13, 14] or in the Gulf of Mexico [15]. The studies are either descriptive epidemiological in nature, or case series with a focus on the causal and contributing factors to accidents. The findings are generally consistent, and they identify for example pilot errors as causal factors in 50% to 70% of helicopter accidents. They also identify technical failures, and in some cases organizational shortcoming that contribute to accidents. For example, Baker et al [11] uncovered that the majority of component failures were due to metal fatigue, not corrosion. Improper pre-flight checks and planning were also identified as important contributors to accidents.

The study of helicopter crashes in Hawaii is of particular relevance for our work. Although the authors worked with a small sample of crashes (59) and only considered a single usage of helicopter (sightseeing tours), they identified improper maintenance as a cause of accident in 31% of the cases (95% confidence interval: 23%–39%). Similarly, Rashid et al. [16] examined 58 maintenance-related helicopter accidents. They analyzed the severity of these accidents, and identified the subsystems most commonly involved in these events (e.g., main rotor, 32%; tail rotor, 17%; engines, 17%). Two interesting findings in their work are (1) inspection’s inability to capture maintenance errors under certain conditions, thus “dramatically annulling” its relevance as a safety barrier in the process, and the existence of (2) “traces of […] over-reliance on, [and expectation that] high-quality and correct work [is] done by maintainers”.

More detail on the helicopter safety literature can be found in the companion article [1]. Our present work is in the spirit of those by Haaland et al. [11], Rashid et al. [16], and Rao et al. [17]. It builds and extends on these works, and it examines some additional dimensions to the problem, including the temporal signature and incubation period from maintenance errors to maintenance-related accidents.

## Data and method

The present work uses civil helicopter accident data publicly available from the National Transportation Safety Board (NTSB) and the Federal Aviation Administration (FAA). We restricted the time span of our analysis to January 2005 till December 2015. We also removed experimental, homebuilt helicopters and gyrocopters from the dataset since these have different airworthiness specifications and special regulations for maintenance and usage. Furthermore, we only included adverse events formally classified as *accidents*. The NTSB defines an *accident* as: (1) an occurrence associated with the operation of an aircraft which takes place between the time any person boards the aircraft with the intention of flight and when all such passengers have disembarked; (2) in which any person suffers death or serious injury; (3) in which the aircraft receives substantial damage. *Incidents*, which result in light or no damage, were not included in this analysis. They offer, nonetheless, a fruitful venue for future work.

The result of our data collection and filtering is the following:

- A dataset with 1,628 helicopter accidents which occurred during this period (2005–2015);
- The data for flight-hours from inspection to accident is available for 698 cases (43% of all accidents). There was no explanation why this data was not recorded for the remaining accidents. Our analysis is therefore enabled by and confined to these 698 cases.

We carefully examined all 698 accident reports. Many of the NTSB accident investigation reports included the following statement, “NTSB investigators may not have traveled in support of this investigation and used data provided by various sources to prepare this aircraft accident report” [8]. The implication is that, as with any study involving data analysis, the limitations due to the quality of the data collected have to be acknowledged. In our case, the dataset we examined is a sample of helicopter accidents over the time period of interest (2005–2015), not the entire population. Although this is a large sample, we cannot gauge whether it is a *convenience sample* or a *random sample*. The implications for our statistical analyses will be discussed later in the text. For all the accident reports, we carefully examined the narratives provided, and we created our own categories and dataset when maintenance and inspections were involved for further analysis. These are discussed in the next section.

Finally, the FAA mandates periodic maintenance and inspection of helicopters to ensure their continued airworthiness. Inspections types and requirements are regulated under the Code of Federal Regulations (CFR), Part 91 for General Aviation and non-commercial transport aircraft, Part 133 for external load helicopters, Part 135 for commercial aircraft, and Part 137 for aircraft used for agriculture. The details and differences are of minor relevance for our purposes. For brevity, we will restrict the description that follows to the requirements in Part 91, under which the majority of helicopters operate.

The Code of Federal Regulation has a complex structure, with many exceptions and interconnections between rules. Title 14, also known as the Federal Aviation Regulations (FAR), is the broadest category that governs all “Aeronautics and Space” activities. The *Parts* are subcategories of Title 14 and they govern a host of topics, vehicles, and requirements. For example, Part 25 details airworthiness standards for the transport category airplanes; Part 61 sets the certification requirements for pilots, flight instructors, and ground instructors; and Part 105 addresses parachute operations.

Part 91, entitled “General Operating and Flight Rules”, also has a tree-like structure and it addresses in its Subpart E, which covers nine paragraphs (from §91.401 to §91.499), rules governing “Maintenance, Preventive Maintenance, and Alterations”. We provide next a couple of examples of the content and language of FAR §91.405 and §91.409. The details that follow may be skipped (the italicized text); nonetheless, we provide them for the interested reader who might be curious about some of the rules and regulations that govern the aspects of aviation of interest to this work:

*Each owner or operator of an aircraft -*
  *(a) Shall have that aircraft inspected as prescribed in subpart E of this part and shall between required inspections*, *except as provided in paragraph (c) of this section*, *have discrepancies repaired as prescribed in part 43 of this chapter*;
  *(b) Shall ensure that maintenance personnel make appropriate entries in the aircraft maintenance records indicating the aircraft has been approved for return to service*;
  *(c) Shall have any inoperative instrument or item of equipment*, *permitted to be inoperative by § 91*.*213(d)(2) of this part*, *repaired*, *replaced*, *removed*, *or inspected at the next required inspection*;
  *(d) When listed discrepancies include inoperative instruments or equipment*, *shall ensure that a placard has been installed as required by § 43*.*11 of this chapter* [Title].

Part 43, which is referenced in §91.405(d), provides a detailed discussion of maintenance and inspection requirements for specific equipment and aircraft parts. Within Subpart E of Part 91, §91.409 entitled “Inspections” covers some of the main issues of interest to this work.

*(a) Except as provided in paragraph (c) of this section*, *no person may operate an aircraft unless*, *within the preceding 12 calendar months*, *it has had -*
  *(1) An annual inspection in accordance with part 43 of this chapter and has been approved for return to service by a person authorized by § 43*.*7 of this chapter; or*
  *(2) An inspection for the issuance of an airworthiness certificate in accordance with part 21 of this chapter [*…*]*
*(b) Except as provided in paragraph (c) of this section*, *no person may operate an aircraft carrying any person […] unless within the preceding 100 hours of time in service the aircraft has received an annual or 100-hour inspection and been approved for return to service in accordance with part 43 of this chapter […]*.

Some exceptions apply, as alluded to in the opening sentence of §91.409. Their details are outside the scope of our work (e.g., aircraft with experimental or light-sport certificates). However, one exception to the entries (a) and (b) is important to note. It relates to the possible substitution of the Annual and 100-hour inspections by Progressive Inspection, described next:

*(d) Progressive inspection*. *Each registered owner or operator of an aircraft desiring to use a progressive inspection program […] shall provide -*
  *(1) A certificated mechanic holding an inspection authorization*, *a certificated airframe repair station*, *or the manufacturer of the aircraft to supervise or conduct the progressive inspection*;
  *(2) A current inspection procedures manual available and readily understandable to pilot and maintenance personnel containing*, *in detail -*
  *(i) An explanation of the progressive inspection*, *including the continuity of inspection responsibility*, *the making of reports*, *and the keeping of records and technical reference material*;
  *(ii) An inspection schedule*, *specifying the intervals in hours or days when routine and detailed inspections will be performed and including instructions for exceeding an inspection interval by not more than 10 hours while en route and for changing an inspection interval because of service experience; […]*;
  *The frequency and detail of the progressive inspection shall provide for the complete inspection of the aircraft within each 12 calendar months and be consistent with the manufacturer's recommendations*, *field service experience*, *and the kind of operation in which the aircraft is engaged*. *[…]*.

In short, four categories of maintenance and inspections are most frequently used for rotorcraft, **(1) the 100-hour, (2) the Annual,** and two types of progressive programs: **(3) the progressive also known as the Approved Aircraft Inspection Program (AAIP), and (4) the Continuous Airworthiness Maintenance Program (CAMP)**. There is little difference between the 100-hour and the Annual, except that the latter requires a technician with an Inspection Authority (IA) to conduct. Whereas the 100-hour can be conducted by any qualified technician, typically an Airframe and Powerplant (A&P) mechanic. An AAIP can be done in lieu of the 100-hour and the Annual, and it contains the same inspection procedures, the only difference being that they are spread over time to minimize downtime given the utilization profile of the rotorcraft [18]. Some additional nuances characterize the CAMP (how it is approved, what Parts it is applicable to, and whether there is fractional ownership of the aircraft), but the details are not relevant for our purposes.

## Results and discussion

**Fig 1:**
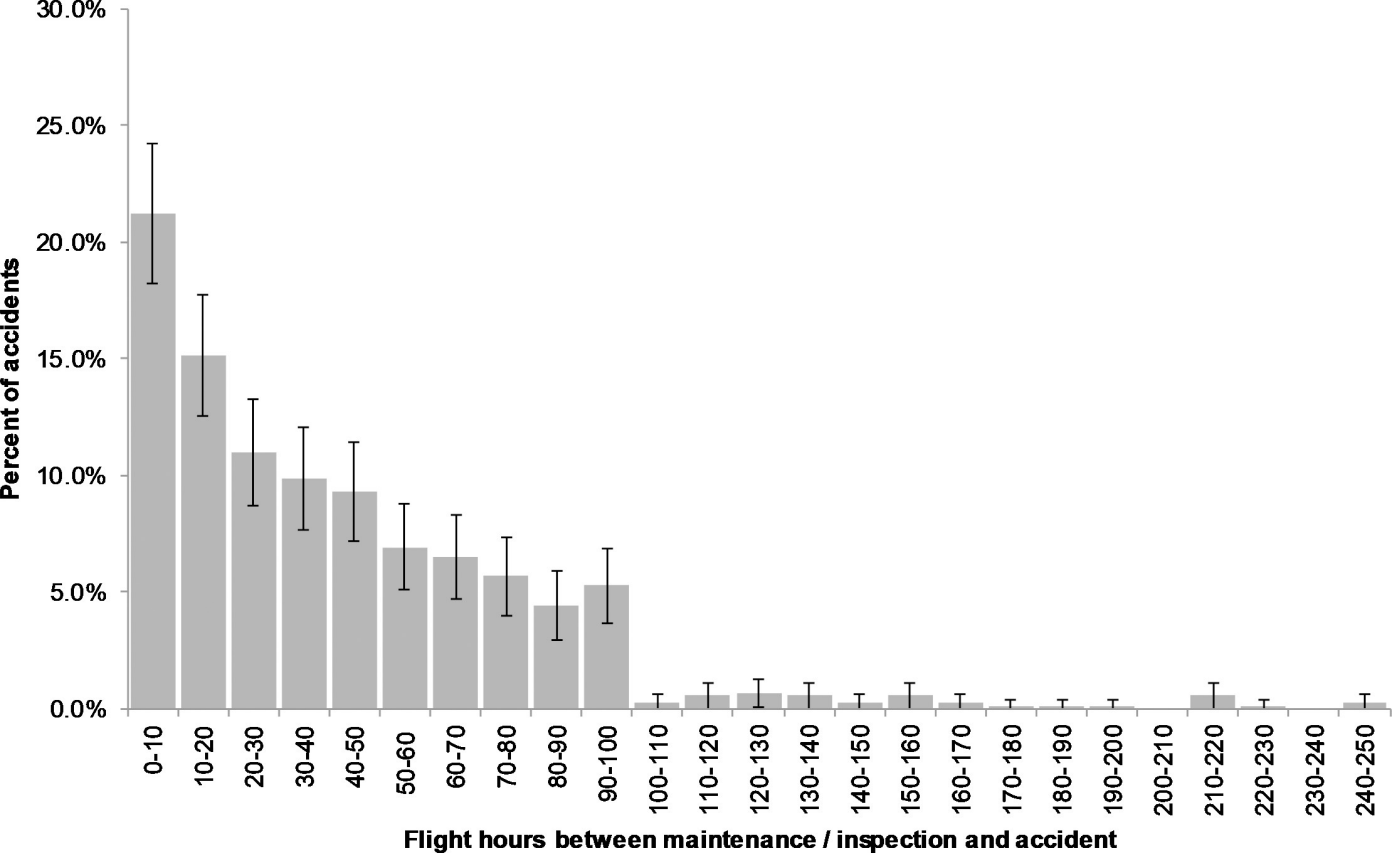
Distribution of flight-hours between helicopter maintenance / inspection and accident (n = 698).

We begin this section with the answer to the original question that prompted this work, namely: what are the features and profile of the *time to accident* of helicopters after maintenance and inspection? Fig 1 provides the results for helicopter accidents in our sample (2005–2015).

Three salient features of these results are the following:

- **There is a clear and significant clustering of helicopter accidents immediately following maintenance and inspection**. For example, about 21% of all accidents occur in the first ten hours of flight following maintenance and inspection, the majority of which occurs within the first couple of hours (the 95% confidence interval covers roughly the 18% to 24% range);
- There is a clear, decreasing pattern of accidents following maintenance and inspection. That is, the likelihood of a helicopter to experience an accident decreases with flight hours after maintenance and inspection. This is reminiscent of *infant mortality* in reliability engineering. The accident trend then levels off around 70 hours. This is reminiscent of the *constant* (*random) failure* rate phase in reliability engineering and the bathtub curve model [19];
- There is a sharp drop in accidents after 100 hours. This is simply an indication of the prevalence of the 100-hour inspection of helicopters in the sample, compared with AAIP and CAMP. All the accidents in Fig 1 after 100 hours are helicopters operating under one of the progressive inspections regime. It is likely that a majority of helicopters in the U.S. civil fleet subscribe to this regime (unfortunately, no official data is available to confirm or improve this estimate).

While Fig 1 provides compelling support for the idea of an association between maintenance and inspection and accidents, caution is required not to jump to conclusions and interpret this association as causation. The following analyses will clarify this issue.

### 4.1 Prevalence of maintenance-related accidents

**Fig 2:**
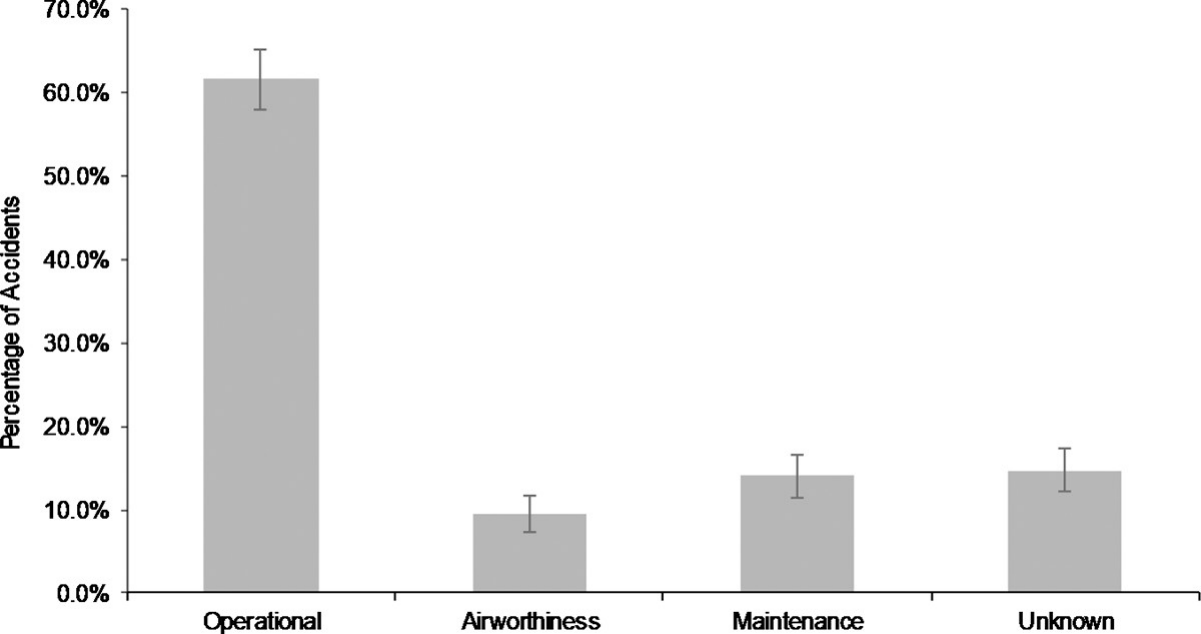
Classification of helicopter accidents by root cause (n = 698).

To further explore this association, we carefully examined all 698 helicopter accident reports in our sample. The results of our analyses are provided next. First, we classified the causes of these accidents, based on the NTSB findings, into four categories, as done in Atkinson and Irving [10]: ***operational***, which includes pilot error and poor pre-flight planning; ***airworthiness***, which includes failure through fatigue, corrosion, or wear of components before their safe life has been exceeded; ***maintenance***, which includes different kinds of maintenance and inspection errors, and ***unknown***, which includes cases the NTSB could not resolve or identify the causes of. The results are provided in Fig 2.

Maintenance and inspection have been identified in the NTSB reports as causes of 14% of all helicopter accidents. The terms “causes” and “causal factors” are used hereafter as determined the NTSB accident investigators through engineering analyses, not in a statistical sense, which would require randomized control trials to ascertain and is obviously impossible in accident analysis. We will refer to these accidents for shorthand as *maintenance accidents*. A further 15% of accidents could not be resolved and have an unknown causal basis. The majority of accidents, 62%, are ascribed to pilot errors.

In some cases, a post-crash fire may have consumed the helicopter and there is little of it left to thoroughly investigate the accident (loss of *material* evidence). This problem is compounded by the absence of black boxes on the majority of helicopters. The implication for our purposes is that some causes of accidents under the *unknown* category may in fact be maintenance accidents. As we discuss next, the 14% maintenance and inspection causes have been clearly identified in the reports, and as a result, **we propose that this 14% constitute a lower bound on the extent to which maintenance and inspection are causal factors in helicopter accidents**.

Two documents are worth mentioning in this regard. The first is an NTSB presentation entitled, “The role of maintenance and inspection in accident prevention” [20, 21]. The document is a case study of a loss of control of a Eurocopter AS350 in Las Vegas on December 7, 2011 in which the pilot and four passengers were killed and the helicopter destroyed. The NTSB identified inadequate maintenance and inspection as the primary cause of the accident. More specifically, the Board identified: (1) the improper reuse of a degraded self-locking nut, (2) the lack of installation of a split pin, and (3) the inadequate post-maintenance inspection, which resulted in a critical flight control unit to separate and render the helicopter uncontrollable. Although this document relates a single case study, it is meaningful in that it identifies one particular failure mode in helicopter accidents and clearly assigns a causal role to (faulty) maintenance and inspection. A discussion of the advantages and limitations of cases studies/series as a methodological approach to accident prevention can be found in the companion article [22].

The second document is entitled, “Power losses on Hughes/MD 500/600 Series helicopters” [23]. This was an internal study by three large helicopter operators and users (A&P Helicopters, Wilson Construction Co, and Winco Inc). The authors examined accidents with these two types of helicopters over an extended period from 1982 to 2014. Of the 182 accidents reported, they found that 53 (29%) were the direct result of ***maintenance errors*, which they defined as “errors in the maintenance of an otherwise airworthy aircraft that caused or significantly contributed to an accident**”. The authors identified several high-level *failure modes* in maintenance errors, in particular: (1) incomplete assembly/installation; (2) incorrect assembly/installation; (3) improper modification / wrong parts used; and (4) Foreign Object Damage (FOD) in the engine, the gearbox, and the compressor. Keeping in mind that the study was limited to these two helicopter series, the MD 500 and 600, and that it extends back over three decades, the results do not necessarily reflect the current situation nor does it necessarily extend to other types of helicopters in the U.S. civil fleet (the MD 500/600 are turboshaft helicopters, and some have gotten rid of the conventional tail rotor in favor of the NOTAR anti-torque system. These would have different requirements and maintenance practices than, say, helicopters with reciprocating engines and traditional tail rotors). Nevertheless, the study is particularly valuable for the rotorcraft community, and the 29% is a sobering statistic of the possible extent of maintenance errors as causal factors in helicopter accidents.

We recommend nonetheless that the NTSB include in its investigation template a section on maintenance and train its investigators to look into this issue.

### 4.2 Temporal signature and incubation period: from *maintenance error* to *maintenance accident*

**Fig 3:**
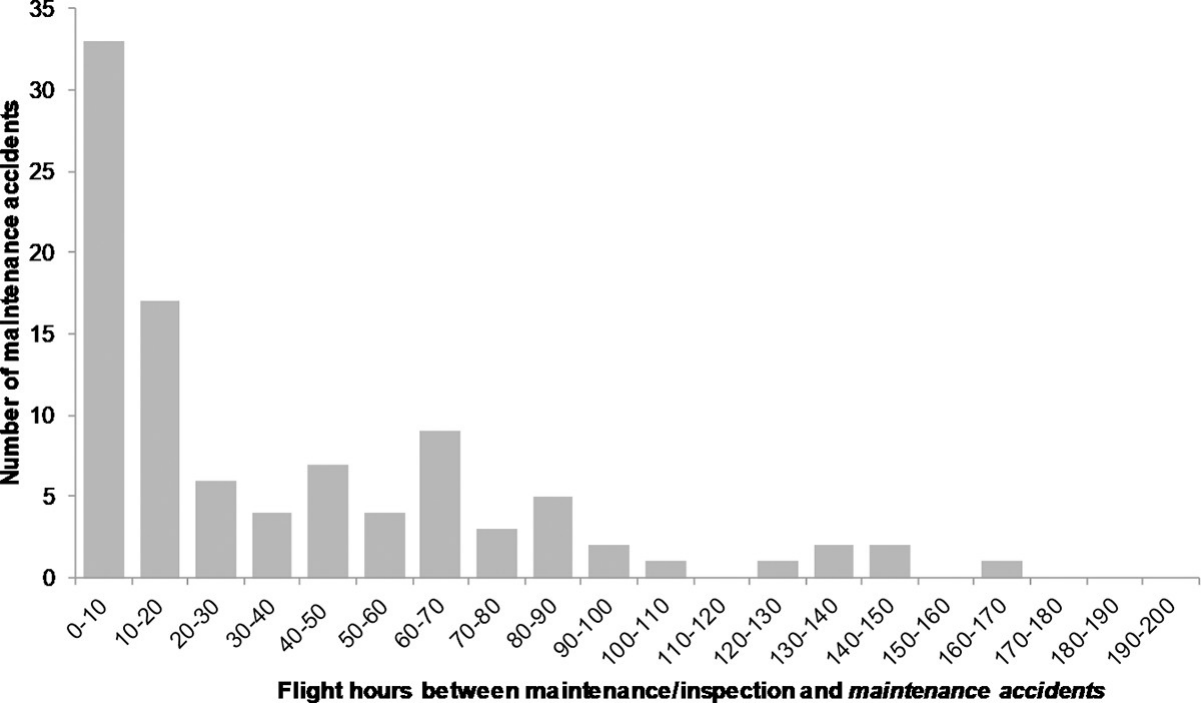
Flight-hours between maintenance/inspection and *maintenance accidents* (n = 98). One case occurred after 200 flight-hours and is not shown for visual clarity.

Following the analysis in Fig 1, our expectations were that maintenance errors turn into accidents immediately after maintenance and inspection are performed on a helicopter. Our analysis of the *maintenance accidents* previously identified proved otherwise. Fig 3 provides the histogram of flight-hours for only the *maintenance accidents*. The histograms for the other categories are provided in the appendix.

The salient features of these results are as follows:

- Many maintenance errors turn into accidents within a short number of flight-hours after maintenance is performed on the helicopter: 34% within the first 10 flight-hours, and 51% within the first 20 flight-hours. When examined with a finer temporal resolution, we find that the first quartile (25%) of maintenance accidents occurs within the first 5 flight-hours;
- The conversion of maintenance errors into accidents decreases with time. This again is reminiscent of infant mortality in reliability engineering, and it can be characterized as the *maintenance error infant mortality*;
- The overall pattern of maintenance accident with time (flight-hours) is more akin to the roller-coaster curve in reliability engineering [24] than the bathtub curve [25];
- Equally important however, is the fact that the “time signature” of maintenance accidents is not confined to the immediate temporal vicinity following maintenance and inspection. We find, for example, that about half of the maintenance accidents occurred after 20 flight-hours, and that the last quartile of such accident occurred after over 60 flight-hours.

How are we to conceptualize these findings? Why do maintenance accidents occur in this temporal manner? **Maintenance errors** can be conceived of **as accident pathogens** injected into a helicopter. After an incubation time or flight-hours, they transform into accidents. An accident pathogen is an adverse latent condition in a system, which, like an infection, when given enough time to incubate or when compounded with other factors or triggers, can precipitate an accident or aggravate its consequences [26]. For example, consider a nuclear power plant with a failed emergency power system. This condition is an accident pathogen in the sense that should the main power system fails, this latent adverse condition will become active and precipitate the accident and lead to a core meltdown [27]. The transition from *maintenance error* to *maintenance accident* is somewhat similar to the evolution of an accident pathogen into a realized accident. Fig 3 shows that this transition can occur over a short period of time; these are maintenance errors that are unforgiving and offer little or no grace period, and they are prevalent among maintenance errors (51% in the first 20 flight-hours). We characterized this feature earlier as the ***maintenance error infant mortality***. **But the transition from maintenance error to maintenance accident can also occur over longer periods of time with maintenance errors (pathogens) slowly metamorphosing into accidents**. For example, we saw previously that the last quartile of maintenance accidents occurs after 60 flight-hours. Is there a physics of failure underlying such differences in incubation time of maintenance errors before they turn into accidents? We provide next examples from the accident reports to illustrate this issue. We begin with cases of maintenance error infant mortality, followed by cases of longer incubation time from maintenance error to maintenance accident.

#### 4.2.1 Case 1: NTSB identification WPR10FA371

The accident occurred on July 28, 2010 in Tucson, AZ, one flight-hour after maintenance and inspection were performed on the helicopter (Eurocopter, now Airbus AS350 B3). Three individuals were killed in the accident. We provide next a few select snippets from the accident report as they provide a rich context for the reader to properly understand and appreciate this problem:

**Phenomenology of the accident**: *About 6 minutes into the flight*, *cruising at 800 feet above ground level*, *the helicopter experienced a complete loss of engine power*. *Witnesses observed the helicopter […] suddenly descend rapidly into a densely populated residential area*. *Descent rates […] were consistent with an autorotation*. *A post-impact fire consumed the cabin and main fuselage of the helicopter*.
**Immediate cause**: *External examination of the engine at the accident site revealed that the fuel inlet union that connected to the fuel injection manifold and provided fuel from the hydro-mechanical unit to the combustion section had become detached from the boss on the compressor case*. *The two attachment bolts and associated nuts were not present on the union flange nor were they located within the helicopter wreckage debris*. *Separation of the fuel inlet union from the fuel injection manifold interrupted the supply of fuel to the engine and resulted in a loss of engine power*. *Post-accident engine runs performed with an exemplar engine showed that*, *with loose attachment bolts and nuts*, *the union initially remained installed and fuel would not immediately leak*. ***As the engine continued to operate*, *the loose nuts would progressively unscrew themselves from the bolts***. *With the bolts removed*, *the union would ultimately eject from the boss*, *and the engine would lose power due to fuel starvation*.
**Maintenance and inspection**: *The repair station technician disassembled [the accident engine]*, *replaced the fuel injection manifold*, *and then reassembled the engine*. *This work required that the fuel inlet union be removed and reinstalled*. *It is likely that the technician did not tighten the bolts and nuts securing the union with a torque wrench and only finger tightened them*. *[…]*. *The repair station technician was serving as both mechanic and inspector*, *and he inspected his own work*.
**Conclusion**: *The National Transportation Safety Board determines the probable cause(s) of this accident as follows*: ***The repair station technician did not properly install the fuel inlet union during reassembly of the engine; the operator’s maintenance personnel did not adequately inspect the technician's work****; and the pilot who performed the post-maintenance check flight did not follow the helicopter manufacturer's procedures*. *Also causal were the lack of requirements by the Federal Aviation Administration*, *the operator*, *and the repair station for an independent inspection of the work performed by the technician*.

This case brings out a host of important issues, from the regulatory and organizational shortcomings in the causal chain of this accident to the technical, immediate factor leading to it, that is, the improper installation and torquing of the fuel inlet nuts, which in turn led to fuel starvation and loss of engine power. This case clarifies one such failure mechanism: in the vibrating environment of the helicopter in operation, the loose nuts progressively unscrewed themselves. The incubation time in this case, one flight-hour, was therefore contingent on several stochastic factors, the extent to which the nuts were improperly torqued and the intensity of vibration in this particular helicopter.

#### 4.2.2 Case 2: NTSB Identification WPR14LA084

This accident occurred on January 1, 2014 in Boulder City, NV, less than one flight-hour after maintenance and inspection were performed on the helicopter (Eurocopter EC 130 B4). No fatalities resulted from this accident. We provide next a brief description of the accident.

**Phenomenology of the accident**: *During a post-maintenance operational check flight and while on final approach for landing*, *the fuel pressure indicator light illuminated*, *and the engine “flamed out*.*” The pilot lowered the collective to initiate an autorotation; however*, *due to the low altitude and airspeed*, *the helicopter subsequently landed hard and rolled over*.
**Maintenance and inspection**: *During a post-accident examination of the wreckage*, *a main fuel supply line B-nut fitting was found without the safety wire*, *and the nut was loose when turned by hand*. *Before the accident flight*, *the line had been disconnected during a task to replace the bidirectional suspension cross-bar assembly*, *and the accident flight was the first flight since the task was performed*. *According to the non-certificated maintenance technician who performed the task*, *the line was removed to defuel the fuel tank*, *which was contrary to manufacturer’s maintenance manual instructions*.
**Conclusion**: *A loss of engine power due to fuel starvation as a result of the non-certificated maintenance technician’s failure to properly tighten and safety wire a B-nut fitting*. *Contributing to the accident was the maintenance technician’s failure to follow the manufacturer’s maintenance manual instructions*.

This accident is similar to the previous one. It highlights three violations as well as significantly poor training and lack of oversight: a non-certified maintenance individual performing this critical task, non-compliance with the manufacturer’s instruction for de-fueling, and most importantly the incorrect and incomplete installation of the B-nut. This reflects a considerably poor safety culture at all levels in this organization. The B-nut is standard terminology for nuts used to connect fluid lines. It is a simple component, but it provides a critical function as a reliable seal in plumbing systems on aircraft and rotorcraft. It is sometimes connected and disconnected during maintenance and inspection on helicopters, and perhaps its simplicity lulls some individuals into handling it casually, “finger tightening it” instead of properly torquing it (at the manufacturer’s specification) and wire-securing it. We have found this to be a recurrent failure mechanism in maintenance accidents.

#### 4.2.3 Case 3: NTSB Identification CEN14LA048

The accident occurred on November 9, 2013 in Shreveport, LA, less than one flight-hour after maintenance and inspection were performed on the helicopter (Eurocopter, EC135 P1). Three individuals were injured in the accident. The following are short snippets from the accident report:

**Phenomenology of the accident**: *The helicopter departed on a local maintenance test flight to perform a hover test*. *The pilot flew the helicopter toward a nearby field to perform the test and then heard a “pop*,*” and the helicopter subsequently began to spin*. *The pilot attempted to regain control of the helicopter using the anti-torque pedals*, *but they were ineffective*. *[…]*. *The helicopter landed hard and rolled on its right side*.
**Immediate cause**: *Examination of the wreckage found that the anti-torque pedals had separated from the anti-torque levers*. *The attachment hardware was not located in the wreckage or the surrounding area*. *Neither the anti-torque pedals nor the lever attachment holes displayed elongation*, *which is consistent with the hardware bolts not being in place at the time of impact*.
**Maintenance and inspection**: *A review of maintenance logbooks revealed that a mechanic had conducted maintenance on the anti-torque pedals before the accident flight*. *After the accident*, *a parts bag containing bolts similar to the bolts needed to secure the anti-torque pedals was found in the maintenance facility where the maintenance was performed*. *Based on the evidence*, *it is likely that the mechanic reinstalled the anti-torque pedals without the required attachment hardware*, *which allowed the anti-torque pedals to separate from the anti-torque levers during flight and led to the loss of helicopter control*.
**Conclusion**: *The National Transportation Safety Board determines the probable cause(s) of this accident as follows*: *The mechanic's improper installation of the anti-torque pedals*, *which resulted in an in-flight loss of helicopter control*.

This accident highlights a functionally different failure mechanism than the previous two cases, namely the loss of (yaw) control of the helicopter instead of loss of engine power leading to the accident. As the pilot activated the anti-torque pedals, his action had no effect on the tail rotor pitch link since the mechanic failed to connect the pedals to the anti-torque levers. Underlying the maintenance error however is the same problem as in the previous cases, namely the incorrect/incomplete reassembly of a critical part.

#### 4.2.4 Case 4: NTSB identification DEN07FA079

The accident occurred on March 27, 2007 in Ponte Verde Beach, FL, one flight-hour after maintenance and inspection were performed on the helicopter (Robinson R44 II). Two individuals were killed in the accident. The following are short snippets from the accident report.

**Phenomenology of the accident**: *Several witnesses observed the helicopter approximately 200–500 feet above ground level in cruise flight along the coastline […]*. *One witness*, *a former pilot and mechanic*, *reported he observed the helicopter in straight and level flight*, *then heard a change in "rotor noise*, *followed by a bang/pop/twang sound*.*" The helicopter then "snap-rolled" to the left and descended into the terrain in a nose low attitude*.
**Immediate cause**: *Examination of the helicopter's flight control system revealed that the right forward servo to swash-plate push-pull tube joint was disconnected and the attach hardware (bolt*, *lock nut*, *two washers*, *pal nut) was missing*. *[…]*. *Material analysis of the components revealed that only one of the two nuts for the left and right connections were installed*, *and then only finger tight*. *The nut on right servo connection rotated off during flight which allowed the bolt to extract itself and disconnect the servo from the push-pull tube*.
**Maintenance and inspection**: *Prior to the accident flight*, *an inspection*, *which required the push-pull tubes to servo connections to be disassembled*, *was performed on the helicopter […]*. *The mechanic who preformed the inspection*, *stated he forgot to properly secure the hardware for the left and right servo connections*. *The mechanic stated the reasons for the error were the following*: *1*. *He was pulled*,*" in all directions" by company personnel since his arrival at that facility; 2*. *The "reassembly was not opposite of the disassembly*,*" which was a personal maintenance practice he used to eliminate errors; 3*. *Two nights prior to the completion of the inspection and the maintenance test flight*, *the apprentice providing assistance*, *wanted to stay late to finish with the mechanic a certain section of the inspection*. *As a result*, *the mechanic forgot to go back and secure the hardware connecting the two push-pull tube to servo joints; 4*. *The company was understaffed with maintenance personnel*.
**Conclusion**: *The National Transportation Safety Board determines the probable cause(s) of this accident as follows*: *the mechanic's improper installation of the attachment hardware for the servo to swash-plate push-pull tube joint which resulted in a disconnection*, *subsequent loss of control*, *and impact with terrain*. *Contributing factors were the company management's inadequate surveillance and enforcement of maintenance procedures*, *[and] the excessive maintenance workload due to inadequate staffing of maintenance personnel*, *[…]*.

It is difficult not to be affected by this (preventable) accident and the reasons provided, especially since two individuals lost their lives. The failure mechanism in this case is a loss of cyclic control, which resulted from an incomplete reassembly of a most critical system on a helicopter, the swash-plate (and associated links). The organizational factors in this accident are also considerable, and there appears to be a significantly poor safety culture at all levels in this organization.

In all these cases, the maintenance error offered no grace period and transformed almost immediately into accidents. Next, we examine a few select cases in which the incubation time was much longer and maintenance errors turned into accidents after more than 60 flight-hours. We include shorter selections from accident reports than the previous cases.

#### 4.2.5 Case 5: NTSB identification CEN12LA120

The accident occurred on January 1, 2012 in Lohn, TX, 68 flight-hour after maintenance and inspection were performed on the helicopter (Bell–Continental 47G2). Two individuals were seriously injured in the accident. The following is a short snippet from the accident report.

*A post-accident examination of the wreckage revealed that the tail rotor gearbox did not operate when it was rotated*. *[*…*]*. *Examination of the ring and pinion gears revealed that* ***the teeth on the pinion gear were deformed and worn almost down to the tooth root in some locations***. *Wear was observed on the top land and drive faces of the ring gear*. *Wear patterns were noted on the coast faces of the ring and pinion gear teeth*. *The observation of wear on the coast faces of the gear teeth is most probably an indication that* ***the spacing between the gears was too tight as a result of improper alignment during installation*, *causing accelerated wear***. *A logbook entry showed that the helicopter’s last annual inspection was completed before the accident during which the tail rotor gearbox was overhauled and installed*. *The National Transportation Safety Board determines the probable cause(s) of this accident as follows*: *The improper installation of the tail rotor gearbox by maintenance personnel*, *which led to accelerated wear of the ring and pinion gears*, *resulting in the loss of tail rotor effectiveness and subsequent forced landing*.

This case highlights another failure mechanism, also prevalent in our sample, namely the misalignment of rotating parts during reassembly, which in turn leads to excessive wear and tear, and ultimately loss of control (e.g., failure at the tail rotor gear box) or loss of engine power (e.g., failure at the transmission drive shaft). The incubation time in this case, from maintenance error to accident, was a long 68 flight-hours. It was contingent on the extent of misalignment during the reinstallation of the part. A more (or less) egregious misalignment would likely result in an accident in a shorter (longer) period of flight-time. There are examples in our dataset of both situations, from a short 3 flight-hours, e.g., the accident identification WPR14TA236, to 99 flight-hours, e.g., LAX05FA264. This again highlights the stochastic nature of the factors affecting the incubation time from maintenance errors to accident.

#### 4.2.6 Case 6: NTSB identification ANC15LA015

The accident occurred on March 13, 2015 in Anchorage, AK, 141 flight-hour after maintenance and inspection were performed on the helicopter (Airbus AS350 B2). Three individuals on-board survived unscathed. The following is a short snippet from the accident report.

*The pilot reported that*, *while the helicopter was in cruise flight […] he felt a “clunk” in the tail rotor control pedals*. *Immediately thereafter*, *the helicopter began to yaw left*. *The pilot attempted to counteract the yaw by pressing the right tail rotor control pedal up to its forward stop*, *but the helicopter did not respond*. *The pilot […] executed an emergency run-on landing*. *[…]*. *An examination of the helicopter revealed that the tail rotor pitch change spider assembly had fractured into two pieces with rotational scarring present along the fractured surfaces; […]*. *Further examination revealed that the spider assembly failure was consistent with* ***bearing seizure*** (side note: Seizure is a catastrophic failure mode in tribological systems (ball bearings and other interacting surfaces with relative motion). It is characterized by stoppage of motion and can be particularly disruptive, especially if high RPM or high inertia are involved. Different failure mechanisms (or physics of failure) can lead to bearing seizure, the most prevalent is oil starvation or improper lubrication. See for example Wang, 1997). *No evidence of [lubrication] grease was found on the bearing surfaces or the bearing housing*. *A review of maintenance records revealed that*, *about 13 months before the accident*, *the pitch change spider assembly was overhauled by a certified repair station*, *during which the original ball bearing was replaced*. *According to the helicopter manufacturer’s spider assembly overhaul procedures*, *grease was to be applied during the installation of the new bearing*. *[…]*. *The National Transportation Safety Board determines the probable cause(s) of this accident as follows*: ***the failure of the ball bearing within the pitch change spider assembly due to its operation with no grease within the bearing***, *which resulted in the subsequent fracture of the spider assembly and a loss of tail rotor control authority*. *Also causal to the accident were* ***the overhaul facility’s failure to follow the helicopter manufacturer’s spider assembly overhaul procedures***, *which resulted in the assembly leaving the facility with no grease in the bearing*, *and* ***the mechanic’s failure to complete all of the tasks on the inspection checklist***, *which led to the lack of grease in the bearing going undetected*.

This case highlights yet another failure mechanism in maintenance accidents. Unlike the previous examples of the improper or incomplete assembly of critical equipment for example, tasks performed incorrectly, this example identifies a “task not performed” when it was required as the maintenance error. The incubation time in this case, from maintenance error to accident, was a long 144 flight-hours. It is perhaps to the credit of the safety margin in the design of the bearing/spider assembly that the system operated for this long before failing.

There are other examples in our dataset in which failure to apply lubrication when required was a causal factor in the accidents, and the corresponding incubation times significantly shorter than the one previously discussed, for example the ERA12FA563 case in which the maintenance personnel applied a corrosion-inhibiting material on critical rotating parts but thinking it was a lubricant (it was not). The maintenance error in this case offered no grace period and resulted in a fatal accident within one flight-hour. As with some of the previous examples, there was non-compliance with helicopter manufacturer's maintenance manual in this case as well as failures at several levels in the organizations, which allowed a non-lubricant to be assessed, purchased, and used (inadvertently) instead of a lubricant for critical parts on the helicopter.

### 4.3 Maintenance and inspection type preceding accidents

**Fig 4:**
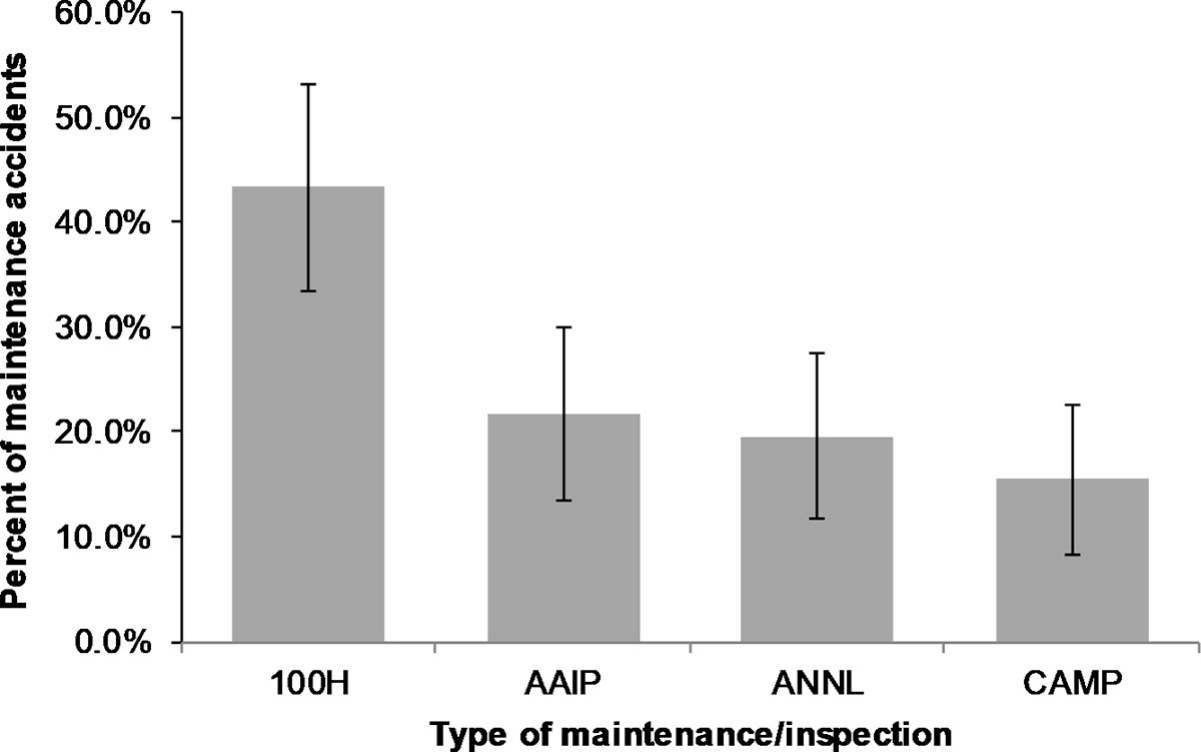
Type of maintenance / inspection preceding a maintenance accident (n = 98).

For all the maintenance accidents, we examined the maintenance and inspection type that preceded each accident. Given our sample and data filters, recall we are concerned with four types: (1) the 100-hour, (2) the Annual, and two types of progressive programs, (3) the progressive also known as the Approved Aircraft Inspection Program (AAIP), and (4) the Continuous Airworthiness Maintenance Program (CAMP). The results are provided in Fig 4. The 100-hour inspection preceded 43% of all maintenance accidents, whereas the progressive programs preceded 21% and 15% of all such accidents for the AAIP and the CAMP respectively.

Caution is advised when considering these results. They cannot be interpreted to mean that one type of maintenance and inspection is associated with more or fewer accidents, since as noted previously, we do not know the extent (percentage) of helicopters in the U.S that are operating under any of these maintenance and inspection regimes. As a result, given this lack of measure of exposure, we cannot establish rates or a hazard index to compare the risks associated with each type of maintenance and inspection leading to an accident. Fig 4 may simply reflect the prevalence of the 100-hour.

If the progressive maintenance programs (AAIP and CAMP) were in the minority, the results in Fig 4 would lead to different interpretations, and ought to prompt a review of these progressive programs, their execution, and their oversight. We encourage the FAA to conduct this analysis (with the prevalence of each type of maintenance) and resolve this issue if the exposure data is available in-house. The outcome can be a negative finding, in which case Fig 4 is a non-issue, or it might lead to a smoking gun and a subsequent safety intervention. Either way this uncertainty is worth resolving.

### 4.4 Helicopter maintenance error classification

A commonly used error classification scheme is the Human Factors Analysis and Classification System, or HFACS, developed in a series of articles by Shappel and Wiegmann [28–30] and based on Reason’s human error classification [31]. The HFACS framework consists of four levels, from the high-level “organizational influences”, to “unsafe supervisions” and “preconditions for unsafe acts”, and down to “unsafe acts” classified as “errors” and “violations”. Each level is further divided into finer-grained categories, for example “errors” are broken down into “decision errors”, “skill-based errors”, and “perceptual errors”, which arise when sensory input is degraded. Decision errors are “[thinking] errors represent [intentional], goal-intended behavior that proceeds as designed, yet the plan proves inadequate or inappropriate for the situation” [29]. Skill-based errors, sometimes described as technique errors, or “doing error”, occur with little or no conscious thought [29]. The classification is reminiscent of the defense-in-depth safety principle and the notion of the safety value chain, individuals at the sharp-end of safety, e.g., operators, and others at the blunt-end of safety, e.g., management [32]. An extension of the HFACS framework was also proposed to cover maintenance-related mishaps in naval aviation [33] and beyond [34]. The levels were specialized to “maintainers condition”, “working conditions” and “maintainer acts”, but little else changed from the original version.

In our sample, the majority of the maintenance errors were skill errors (77%) and very few decision errors (12%). We found no perceptual errors, and the accident narrative was not descriptive enough to assign a particular error type for the remaining errors (11%).

We did not adopt or further pursue the HFACS framework in our work for two reasons. First, except in rare cases, there was no discussion of organizational influences or the pre-conditions for unsafe acts in the accident reports (Level 1 and 3 in HFACS). Furthermore, in the vast majority of cases, poor inspection was at fault (Level 2). Second, more importantly, we found repeated patterns of maintenance errors (by function and location on a helicopter), which the HFACS unsafe acts classification was blind to. We propose the following classification (based on [10]) and hierarchical decomposition instead, which is tailored to our sample and the clusters of maintenance errors we encountered. Our main consideration in developing this was to have something practical for operators and that can help guide safety interventions, as we will see next.

**Fig 5:**
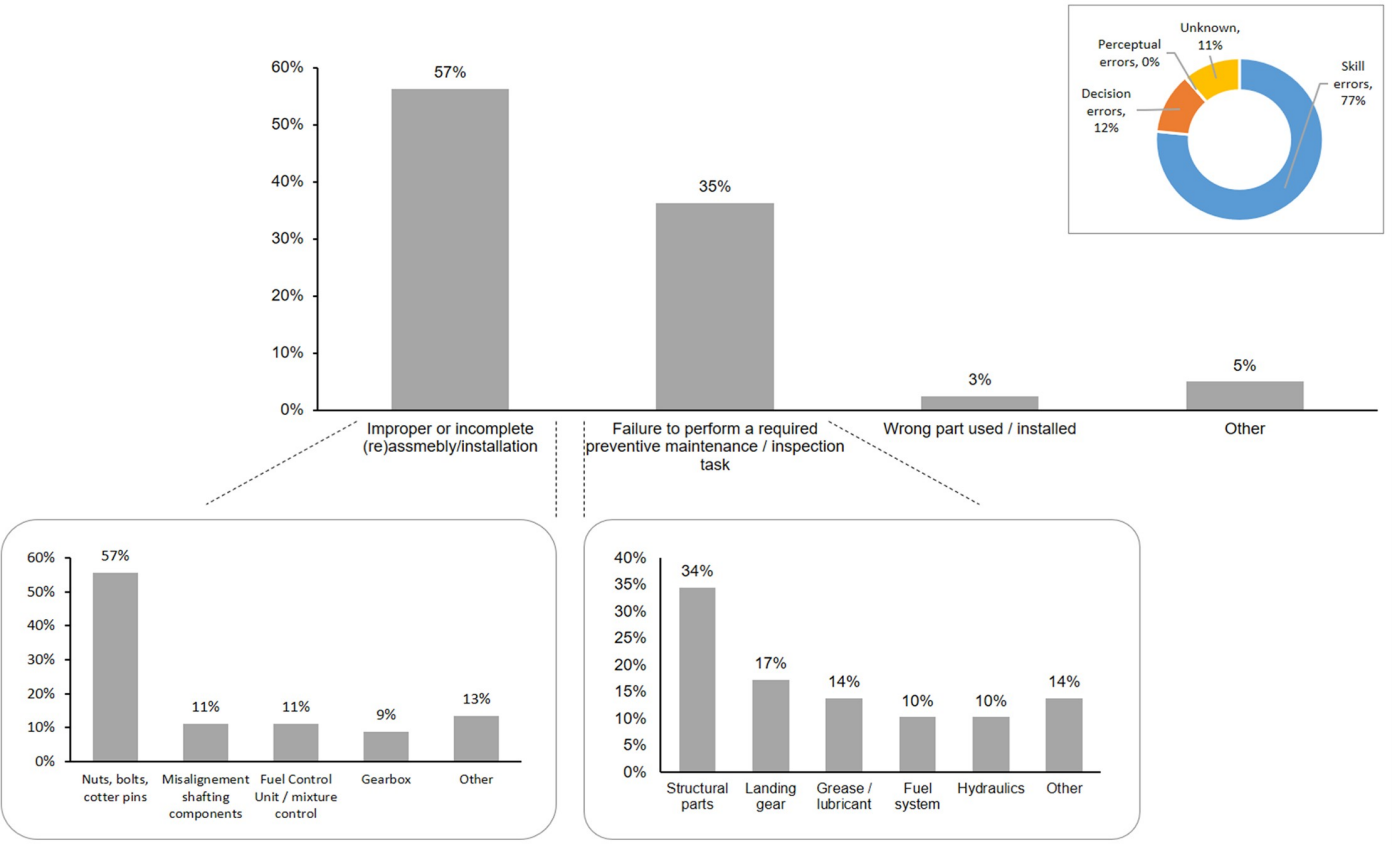
Classification and prevalence of helicopter maintenance errors.

The results are shown in Fig 5. At the top level, we have:

1. *Improper or incomplete (re)assembly or installation of a part*. This accounted for the majority of maintenance errors with 57% of such cases;
2. *Failure to perform a required preventive maintenance and inspection task*: 35%;
3. *Use or installation of the wrong part*: 3%;
4. *Other*. This category accounted for situations we either could not resolve or for which there was only a single occurrence.

We further decomposed these categories when enough data was available in the accident reports. For example, the following sub-categories clearly emerged under the *Improper or incomplete (re)assembly or installation of a part*. We found for example that:

1.1 Incorrect torquing or incomplete assembly of nuts, bolts, cutter pins, or safety wires was the most prevalent sub-category in (1), accounting for 57% of the maintenance errors under this category (1);
1.2 Misalignment of shafting components after maintenance accounted for 11% of this category;
1.3 The incorrect assembly of the fuel control unit and mixture control accounted for another 11% of this category;
1.4 The incorrect assembly of the gearbox accounted for 9% of the maintenance errors in this category.

**Fig 6:**
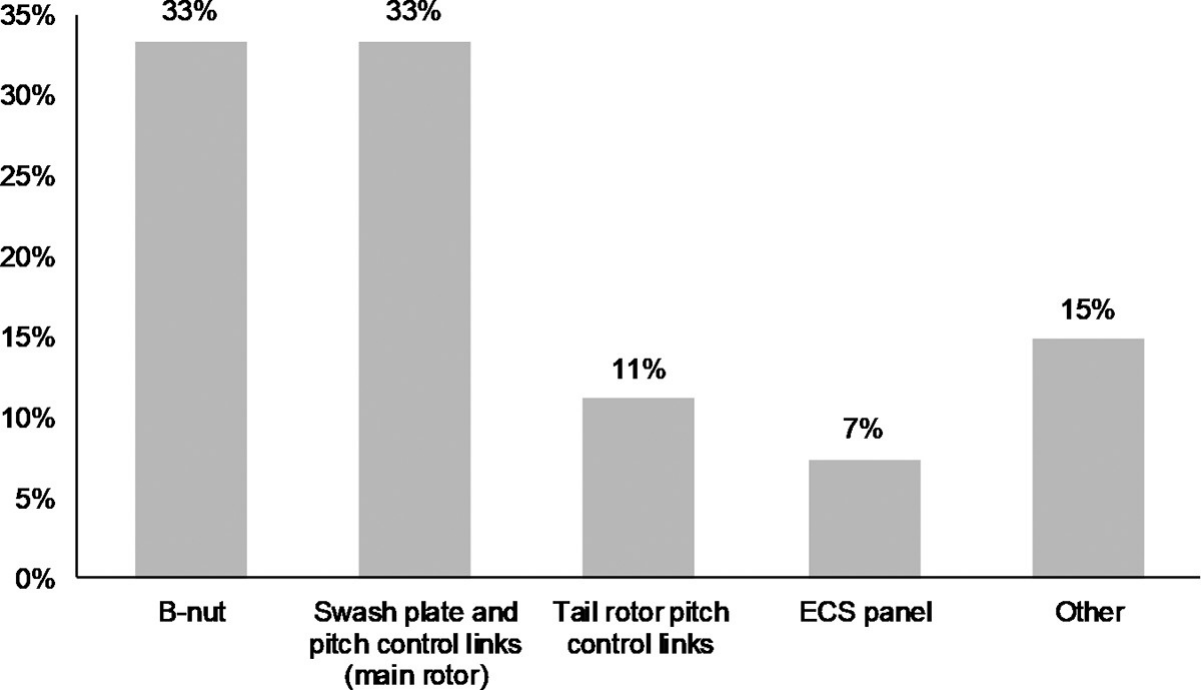
Sub-classification and prevalence of helicopter maintenance errors under the “*Incorrect torquing or incomplete assembly of nuts*, *bolts*, *cutter pins*, *or safety wires”*.

A further sub-classification of (1.1) yielded the following results (see Fig 6):

1.1.1 The B-nut was involved in 33% of the cases of incorrect torquing or incomplete assembly of nuts, bolts, cutter pins, or safety wires;
1.1.2 The swash plate and pitch control links (main rotor) were also involved in 33% of these cases;
1.1.3 The tail rotor pitch control links were involved in 11% of these cases.

Going back to Fig 1, we further decomposed the second category, *Failure to perform a required preventive maintenance and inspection task*, with the parts to which the preventive maintenance was meant to be applied and for which there was enough data. The results show:

2.1 A prevalence of structural parts (rotor blades, structural tubing, engine outer combustion case, upper sheave, etc.) not properly inspected or maintained, 34%. **It is important to clarify that this means that the maintenance program was not executed in compliance with federal regulations, nor with the manufacturer maintenance plan or service bulletins**. In some of these cases, the parts were overflown past their service life by several hundred hours;
2.2 The landing gear was surprisingly involved in 17% cases of maintenance errors in this category. In this sub-category, the maintenance error led to ground resonance, and the consequence was dire: as one (un)lucky pilot was quoted in the accident report, “in a matter of seconds, the helicopter shook itself apart”;
2.3 The non-application of grease or lubricant when required was involved in 14% of the cases in this category. The failure mechanism in these cases typically involved thermal damage, which led to structural failure.

Furthermore, breakdown of these or other (sub-)categories was either impossible or not meaningful at the present and given our sample. It is worth acknowledging that within the HFACS framework, our second category, *failure to perform a required preventive maintenance and inspection task*, can be classified either under *errors* or under *violations*. In a couple of cases, the accident reports specify this was the result of poor record keeping and oversight. For the majority of cases however, no such information is available, and it is understandably difficult to ascertain. Although it is unlikely that there were any willful violations of safety-critical preventive maintenance tasks, our classification avoids this dichotomy in the HFACS classification at Level 4.

Two final comments: the magneto was involved in two cases, each one under a different category, *improper or incomplete (re)assembly*, and *failure to perform*…. As a result it does not appear in Fig 5 (it is under “other” in both cases). This is a blindspot in our classification and further refinement of our scheme is warranted in a few years when more data becomes available. In three cases, the maintenance documentation provided by the manufacturer was either lacking or unclear. In the remaining vast majority of cases however, the maintenance errors were clearly non-compliant with official documentation.

## Defense-in-depth against maintenance errors: Preliminary recommendations

In this section, we propose a set of recommendations for addressing maintenance errors, which are based on the findings in the previous section on the one hand, and which borrow from the ideas underlying the defense-in-depth safety principle on the other hand. First, a brief discussion of this principle is provided.

### 5.1 Defense-in-depth

Defense-in-depth is a fundamental safety principle that is widely adopted in different industries, sometimes under different names but with the same underlying basis [35, 36]. It derives from a long tradition in warfare by virtue of which important positions were protected by multiple safety barriers or lines of defenses (e.g., moat, outer wall, inner wall). First conceptualized in the nuclear industry, defense-in-depth became the basis for risk-informed decisions by the U.S. Nuclear Regulatory Commission [37, 38]. Defense-in-depth has several pillars:

- Multiple lines of defenses or safety barriers should be placed along potential accident sequences;
- Safety should not rely on a single defensive element (hence the “depth” qualifier in defense-in-depth);
- The successive barriers should be diverse in nature and include technical, operational, and organizational safety barriers. In other words, defense-in-depth should not be conceived of as implemented only through physical or technical defenses.

**Fig 7:**
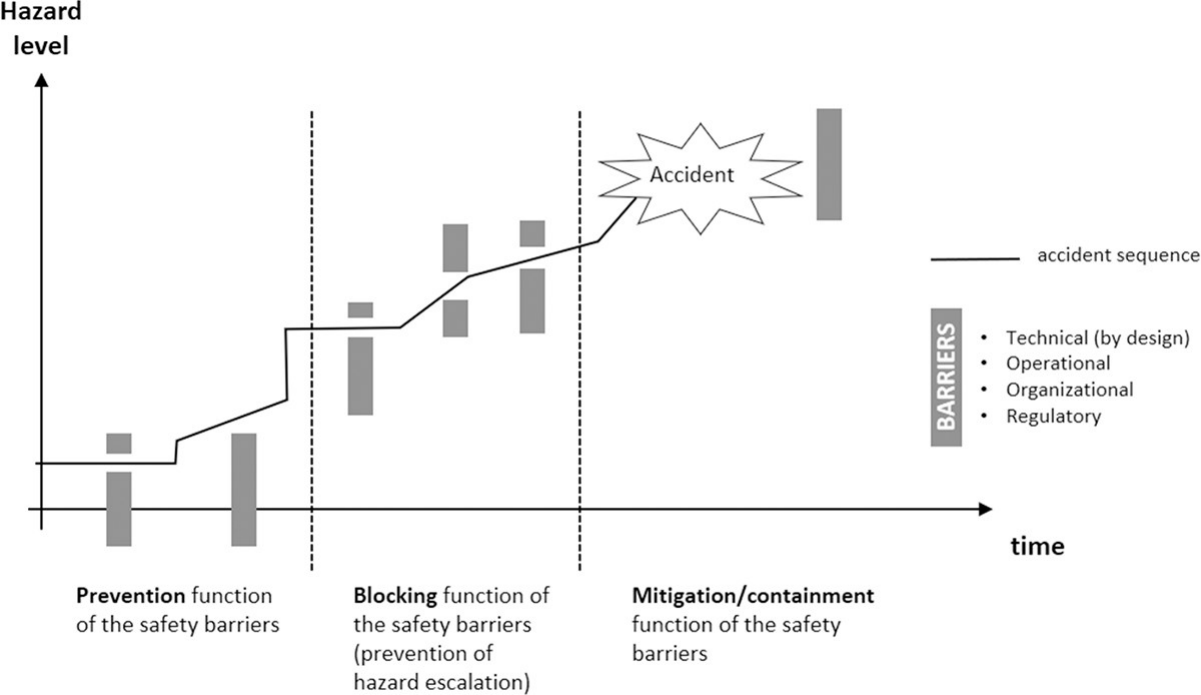
Illustration of the defense-in-depth safety principle, along with a hypothetical accident sequence (the accident occurs as a result of the absence, inadequacy, or breach of various safety barriers).

The various safety barriers have different objectives and perform different functions. The first set of barriers, or line of defense, is meant to prevent an accident sequence from initiating. Should this first line of defense fail in its **prevention** function, a second set of safety defenses should be in place to **block further escalation** of the accident sequence. Finally, the third line of defenses is designed and put in place to minimize or **mitigate the consequences of the accident** should the previous barriers fail in their function. Lifeboats are one illustration of this third line of defense. Crash-resistant fuel systems are another example of this third line of defense for helicopter (post-crash fires have been responsible for fatalities in several helicopter accidents). Fig 7 illustrates this safety principle, along with a particular accident sequence.

### 5.2 Recommendations

The following recommendations, as noted previously, are based on our examinations of the maintenance accidents, and they are informed by the safety principle of defense-in-depth. They include technical, operational, and organizational recommendations. At a high level, the fundamental recommendation is to develop a strong safety culture among individuals and organizations who deal with helicopter maintenance. This was clearly lacking in the majority of accidents examined. Some ways for achieving this given our findings are discussed next.

**R1. Provide better training and certification**: Our results provide a clear indication that better training of maintainers and inspectors would be beneficial. Figs 5 and 6 narrow down the scope of this broad recommendation and identify **critical hot spots conducive to maintenance errors**. These are specifically the tasks and areas that deserve more emphasis for proper training and certification. For example, the repeated improper torquing or incomplete assembly of nuts, bolts, and cutter pins should not be accepted as a common risk in helicopter maintenance; it is easily preventable with better training and a dedicated module to these assemblies (B-nut, swashplate, pitch control links), and some form of verification that these critical skills have been properly retained.
**R2. Develop checklists and emphasize compliance**: Except in rare cases when documentation was lacking or not clear, most maintenance accidents in our sample could have been avoided if the work was done in compliance with federal regulation and manufacturer’s maintenance plan or service bulletins. It is important that operators and regulators repeatedly emphasize the importance of compliance with official documentation, that usable checklists be developed, and that maintenance processes and execution be occasionally audited.
**R3. Strongly communicate the importance and safety-critical nature of maintenance and inspection tasks**: helicopter maintenance is a momentous responsibility, and it cannot be treated with the same attitude as the maintenance of a pedestrian item such as an HVAC or a dishwasher. In several of the cases we examined, it appears that maintenance and inspection were treated with a casual level of attention and without an appreciation for the serious potential consequences down the road. The community would be well served if helicopter operators, regulators, and professional societies developed a communication and outreach campaign to emphasize the safety-critical nature of the work of maintainers and inspectors.

The three previous recommendations operate at the prevention level within a defense-in-depth framework, that is, striving to avoid the initiating event or maintenance error from being committed in the first place. But should this prevention function fail, another set of safety barriers should be in place to catch these accident pathogens and block an accident sequence from further escalating. The following address this part.

**R4. Organize for, and execute careful inspection and quality assurance of all maintenance work**: in the vast majority of cases, poor inspection of maintenance work was noted as a failure to catch the maintenance error (under the first category, *improper or incomplete (re)assembly or installation of a part*). Furthermore, in some cases, either no inspection was conducted to verify the quality of the maintenance work, or the same person who performed the maintenance inspected their own work as well. The pilot was also noted on several occasions to have failed to notice the problems during pre-flight checks. We strongly recommend that operators develop a formal process (if it is not already in place) and assign dedicated quality assurance personnel if they can afford it, to inspect all maintenance work, especially those involving critical bolts and nuts. This need not be a time-consuming activity, even if done thoroughly. Incorrect torquing and assembly of critical bolts and linkages for example, which is responsible for the largest proportion of maintenance accidents, can be easily caught with proper inspection, and this opportunity should not be forfeited for accident prevention.
**R5. Re-examine the conduct of post-maintenance flight check?** This is not a recommendation per se since there was no data in the accident reports to inform it. However, we raise the fact that some accidents occurred immediately after maintenance was performed (in some instances within the first flight-hour). Fatalities also occurred on these flight. As a result, we invite the rotorcraft community, especially pilots and maintainers, to reflect on the following question: are there ways to (re-)design post-maintenance flight checks to tease out some of the maintenance errors we see in Figs 5 and 6? And to do it in a more benign and controlled way without risking lives and limbs?

There was anecdotal evidence in some accident reports that organizational issues were contributing factors to the accident. We propose the following tentative recommendation and recognize the difficulty of operationalizing it. However, we believe this deserves careful consideration, and it may be adopted in different forms given the particular circumstances of each operator.

**R6. Isolate (excessive) flight pressure from maintainers, and carefully manage workload of maintenance personnel.** In some of the accident cases, there was clear maintenance work overload, the maintainer “felt pulled in all directions”, and perhaps thoroughness was sacrificed for availability. Mechanic’s fatigue and long working hours were also noted in the NTSB documents discussed previously as contributing factors to accidents [20, 21]. It should be strongly impressed upon operators that cutting corners on helicopter maintenance is rarely, if ever forgiving, and it is never acceptable. Formal processes should be in place (and abided by) that actively manage the workload of maintenance personnel. And while operational pressure is unavoidable, it is essential that maintainers be empowered to never feel the urge to cut corners or sacrifice safety vigilance.

We conclude with a final recommendation specifically addressed to the Federal Aviation Administration. We urge the FAA to establish a multi-stakeholder taskforce, which would include helicopter manufacturers, operators, pilots, and maintenance personnel to bring their collective wisdom to bear on these issues and devise targeted recommendations and effective safety interventions to address this disquieting problem.

## Conclusion

**Fig 8:**
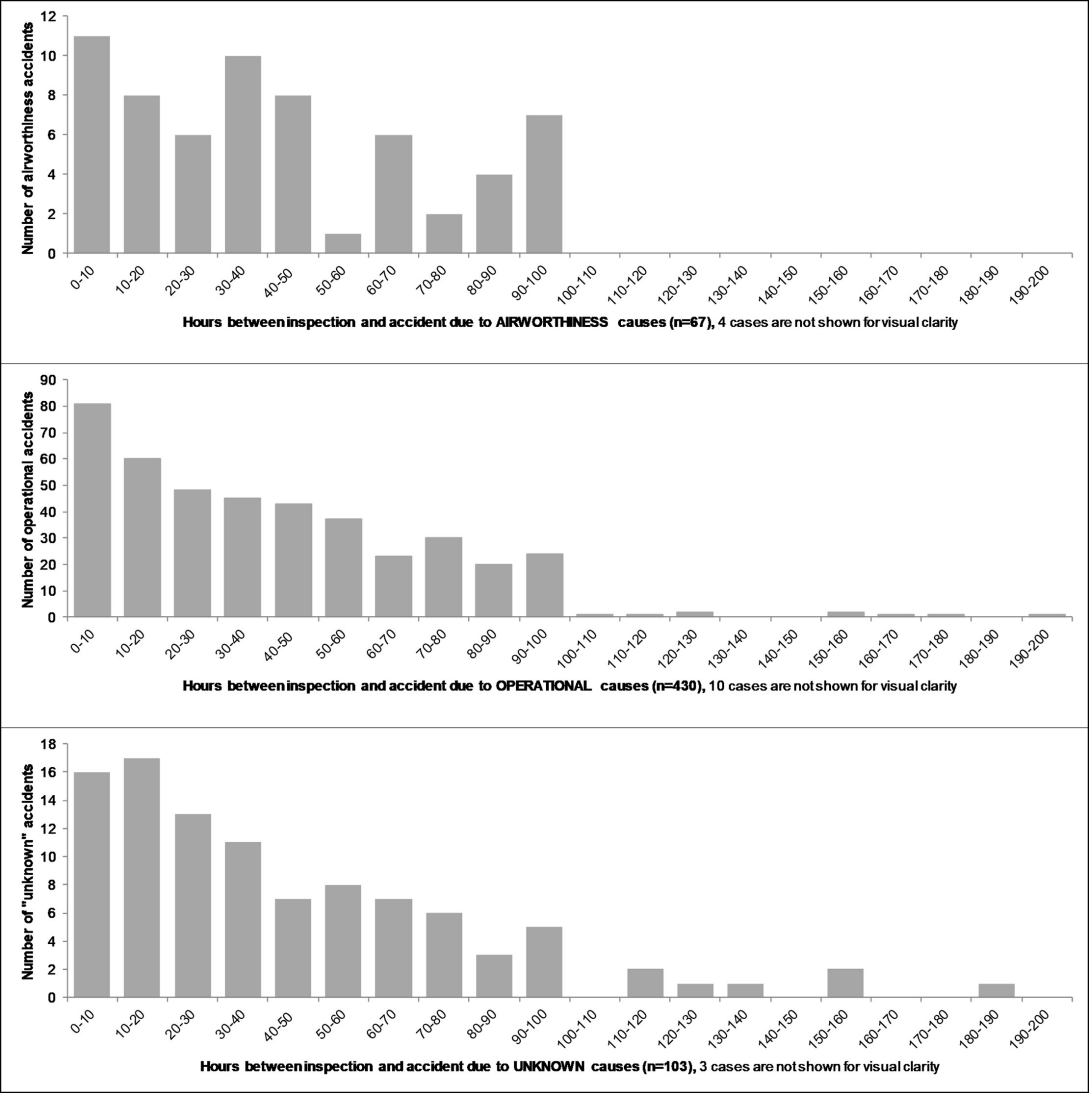
Flight-hours between maintenance/inspection and *accidents* (n = 698).

In this work, we first established that maintenance and inspection were a risk factor in helicopter accidents. Between 2005 and 2015, we found that flawed maintenance and inspection were causal factors in 14% of helicopter accidents in the U.S. civil fleet, excluding homebuilt and experimental helicopters. We argued that this figure constitutes in all likelihood a lower bound, and that the actual extent is closer to 21%. With a rough average of about 150 helicopter accidents per year over the time period here considered, this represents about 20 to 30 helicopter *maintenance accidents* per year. The histograms for the other categories of accidents can be found in the appendix in Fig 8.

For these maintenance accidents, we then examined the incubation time or duration (flight-hours) from when the maintenance error was committed to the time when it resulted in an accident. We found a significant clustering of maintenance accidents within a short number of flight-hours after maintenance and inspection were performed. For example, 31% of these accidents occurred within the first 10 flight-hours. We also found that the conversion of maintenance errors into accidents decreases with time, which is reminiscent of infant mortality in reliability engineering, and we characterized it as *maintenance error infant mortality*. Equally important however was the finding that the “time signature” of maintenance accidents is not confined to the immediate temporal vicinity following maintenance and inspection. We found for example that about half of the maintenance accidents occurred after 20 flight-hours, and that the last quartile of such accidents occurred after 60 flight-hours following maintenance and inspection.

We also examined some of the “physics of failures” underlying maintenance accidents. We then proposed a classification of maintenance errors and analyzed their prevalence in helicopter accidents. We found for instance that the *improper or incomplete (re)assembly or installation of a part* category accounted for the majority of maintenance errors with 57% of such cases, and within this category, the incorrect torquing of the B-nut or incomplete assembly of critical linkages was the most prevalent maintenance errors. We also found that within the *failure to perform a required preventive maintenance and inspection task* category, the majority of the maintenance programs were not executed in compliance with federal regulations, nor with the manufacturer maintenance plan or service bulletins.

Finally, we provided a set of recommendations based on our findings and which borrow from the ideas underlying the defense-in-depth safety principle.

We indicated throughout this work a number of fruitful venues for future work such as, for example, expanding the scope of the analysis to include helicopter incidents from the NTSB database, not just accidents as we have done in this study. This has the potential to yield additional insights, identify precursors to helicopter accidents, and further strengthen our present findings. We also discussed the importance of examining in future work the prevalence of different types of maintenance programs (AAIP and CAMP) and analyzing “maintenance accident rates” associated with each type. These topics, however, are of little significance when compared with what we consider the most important future work, namely the planning and execution of safety interventions to address this problem. Every year, dozens of helicopter accident occurs in which maintenance errors are causal factors, and more individuals are killed or injured in the process. These are preventable accidents, and this heavy cost in lives and limbs need to be paid. We reiterate our most important recommendation, that the FAA establish a multi-stakeholder taskforce, which would include helicopter manufacturers, operators, pilots, and maintenance personnel to bring their collective wisdom to bear on these maintenance issues and devise targeted recommendations and effective safety interventions to eliminate this disquieting problem.

We conclude this work with a paradoxical statement, that helicopter maintenance and inspection are highly reliable. How do we reconcile this statement with the previous analyses? We examined in this work the likelihood that an accident has a maintenance contribution, given that an accident has occurred. The 14% quoted previously is the estimate of the following conditional probability, $\hat{p}(maintenance|accident)$. The current statement relates to the conditional probability that given that maintenance and inspection have been performed on a helicopter, what is the likelihood that it will lead to an accident, or $\hat{p}(accident|maintenance)$. There is no data to carefully assess this quantity. We propose nonetheless the following back-of-the-envelope calculations:

There are roughly 12,000 helicopters in the U.S. fleet, their average flight-hours per year is about 300 hours (these are not uniformly distributed across different types of helicopters). Assuming 90% of helicopters operate under the 100-hour and annual inspection regime, this results in $12,000\times 0.9*\left(\frac{300}{100}+1\right)=43,200$ maintenance and inspection interventions per year. Furthermore, assuming that each of the remaining helicopters operating under the progressive maintenance regimes (AAIP or CAMP) have 6 maintenance and inspections interventions per year each, this would result in an additional 7,200 interventions. As a result, it is reasonable to assume that there are perhaps around 50,000 maintenance and inspections interventions on U.S. civil helicopters per year. With the 20 to 30 helicopter *maintenance accidents* per year noted previously, this give an average reliability of the maintenance and inspection intervention of 99.94% to 99.96%, or 4 to 6 in 10,000 intervention lead to an accident.

Our comment that helicopter maintenance and inspection are highly reliable is related to this estimate. It is nevertheless worthwhile to reflect on whether this is a good reliability achievement to be content with or not. If banks for example had a similar reliability per transaction, the result would likely be financial mayhem and riots. Amalberti *et* al [39] benchmarked approaches to safety in different high-risk industries, and found a typical value of 10^−5^ to 10^−6^ of unreliability per unit of exposure in a healtchare context (blood transfusion and anesthesiology) and in civil aviation (per departure) for example. This represents about 2 to 3 orders of magnitude better reliability than that of helicopter maintenance and inspection. The rotorcraft community deserves and can do better.
